# Nutritional and Functional Status as a Predictor of Short-Term Mortality in Hospitalized Elderly Patients in a Tertiary Care Hospital

**DOI:** 10.7759/cureus.22576

**Published:** 2022-02-24

**Authors:** Megha Mukundan, Kritartha Kashyap, Minakshi Dhar, Aishwarya Muralidharan, Disha Agarwal, Yogesh Saxena

**Affiliations:** 1 Internal Medicine, All India Institute of Medical Sciences, Rishikesh, Rishikesh, IND; 2 Public Health, All India Institute of Medical Sciences, Rishikesh, Rishikesh, IND; 3 Physiology, Swami Rama Himalayan University and Himalayan Hospital, Dehradun, IND

**Keywords:** prognostication, mortality, malnutrition, geriatric population, activities of daily living

## Abstract

Context

Elderly people are at a high risk of malnutrition leading to poor outcomes and quality of life.

Aims

We aimed to find an association between the nutritional and functional status of hospitalized elderly patients and the three-month all-cause mortality among them.

Settings and design

A cross-sectional study was carried out at a tertiary care hospital in North India from July 2018 to December 2019.

Methods and material

A total of 177 patients were recruited for the study, and their demographic and clinical data were collected on a preformed questionnaire. Comorbidity, nutritional status, functional status, and depression were calculated using the Charlson Comorbidity Index (CCI), Mini Nutritional Assessment (MNA) form, Katz Index of Independence in Activities of Daily Living (Katz ADL), and Geriatric Depression Scale (GDS), respectively.

Statistical analysis

A Chi-square test was used to find the association between different qualitative variables. A regression model was used to find out the odds for mortality. Statistical significance was set at p<0.05.

Results

According to the MNA score, 49.7% (88) were at risk of malnutrition, and 22.6%(40) were malnourished. Malnutrition, Charlson Comorbidity Index, and the functional status of the patients were found to be associated with three-month mortality, with a p value of 0.005, 0.017, and 0.021, respectively. On regression analysis, malnutrition (odds ratio (OR): 3.796; 95% confidence interval (CI): 1.178-12.234; p=0.025) and the functional status (OR: 3.160; 95% CI: 1.256-7.952; p=0.015) of the study participants were found to have higher odds for three-month all-cause mortality.

Conclusions

Nutritional status and ADL assessed at the time of discharge are good prognostic markers of health outcomes in the elderly population.

Key message

ADL and nutritional assessment at admission and discharge should be routinely incorporated in the geriatric assessment of hospitalized patients to triage and prognosticate.

## Introduction

The elderly population per se is vulnerable to health hazards, comorbid conditions leading to hospital admission, and often long-term care. It is not unusual to see these patients often succumbing to their ailments regardless of the duration of the hospital stay. A lot of factors have been implicated over the years for this decline in the health of the elderly, and this has been a grave concern among healthcare providers, especially geriatricians. Out of all the attributable causes, nutrition has been a prime concern for the optimal health of older persons. It is an important modifiable index of health and well-being in all age groups [1]. Nutritional decline is very commonly seen among hospitalized elderly and has always been underestimated or underdiagnosed due to misdirected or improper attention. Malnutrition generally occurs when nutrient intake is consistently insufficient to meet individual nutrient requirements, and this imbalance between nutrient intake and requirements ultimately results in changes in body weight, body composition, and physical function [2]. Nutritional decline has been observed to increase the susceptibility to infections, severity of diseases, length of stay in the hospital, duration of complete recovery of the disease process, dependence on multiple daily medications, and dependence in activities of daily living (ADL). It can lead to increased incidence of depression due to health and health-related issues and increased rate of readmissions and can reduce life expectancy [3]. A single standardized score is not yet available for the assessment of nutritional status in the elderly. However, some of the standardized tools commonly used to assess nutrition include the Subjective Global Assessment (SGA), Mini Nutritional Assessment (MNA) and its short form (SF-MNA), Nutrition Risk Screening, and “Malnutrition Universal Screening Tool” (MUST).

At the same time, elderly people often have multiple comorbid conditions, causing a greater impairment in their functional capacity. Hence, decreased functional status is seen as a common presentation of many diseases in the elderly population. Geriatric functional status can be objectively assessed using the Katz Index of Independence in Activities of Daily Living (Katz ADL). Many studies have shown that a greater dysfunction of ADL was independently predictive of mortality in the geriatric age group [4].

Despite their need to be worked upon and their effect on healthcare quality, both nutritional and functional status are most often neglected while assessing an elderly patient. Although a part of comprehensive geriatric assessment, due to a lack of awareness or knowledge, these two important parts of elderly evaluation have long been overlooked. However, both nutritional and functional status have seen a resurgence of importance recently as it can be predictive of morbidity and mortality in elderly patients. A number of studies have been done to study the effect of malnutrition on mortality, but those patients who are at risk of malnutrition have been neglected. Deterioration in both nutritional and functional aspects in the elderly age group has led to a greater healthcare burden in recent times. Geriatricians should play an active role in this aspect and screen these patients during in-hospital stay to find out at-risk populations, so as to improve their nutrition and functional status at the time of discharge for a better quality of life. Thus, this study was planned to determine nutritional and functional status as predictors of three-month all-cause mortality in hospitalized elderly patients.

## Materials and methods

**Figure 1:**
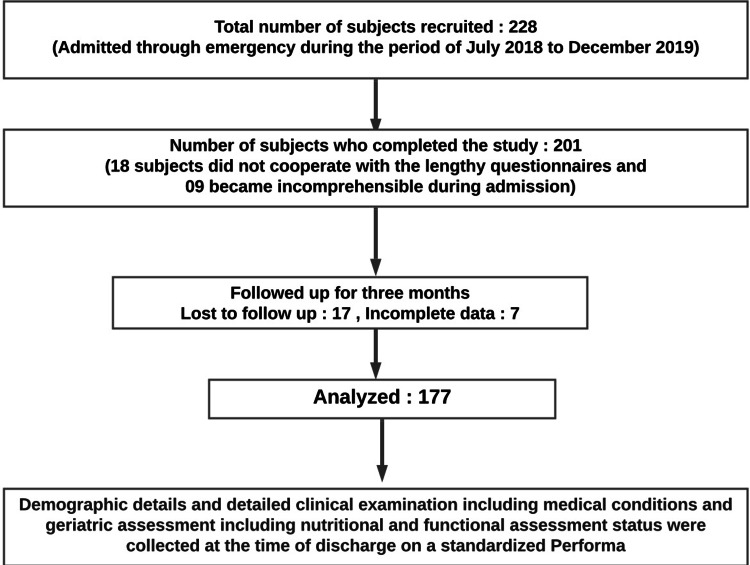
Flowchart of the study methodology

The study was conducted over a period of 18 months from July 2018 to December 2019. The sample size of 177 elderly patients was calculated by taking the prevalence of malnutrition in hospitalized elderly as 36% (based on the study on the prevalence of malnutrition by Middleton et al.) [5] taking 80% as the power of the study, with 95% confidence interval (CI) and 7.2% precision, using the formula 4pq/L [2]. All elderly patients who were admitted to the inpatient department with MMSE scores of more than 25 and gave consent were included in the study. All patients who were incomprehensible and had terminal illnesses were excluded from the study. Demographic details and detailed clinical examination including medical conditions and geriatric assessment including nutritional and functional assessment status were collected at the time of discharge on a standardized performa (Figure 1).

Ethical clearance was obtained from the institutional ethics committee (AIIMS/IEC/18/544). Written informed consent was taken from the participants before commencing the study. The objective of the study was to identify the predictability of nutritional and functional status of elderly patients on their three-month all-cause mortality.

Nutritional assessment was done in all patients at the time of discharge using the Mini Nutritional Assessment (MNA). The MNA is a validated tool useful for assessing nutritional status in the elderly, with a sensitivity of 96%, specificity of 97%, and predictive value of 97% [6]. Patients whose MNA scores came out to be ≥24 were categorized as well-nourished, those with scores < 17 as malnourished, and patients with scores between 17 and 23.5 as at-risk.

Activities of daily living were evaluated using the Katz Index of Independence in Activities of Daily Living (Katz ADL) [7]. The index ranks the adequacy of performance on the following six functions: bathing, dressing, toileting, transferring, continence, and feeding. A score of 6 indicates full function, 4 indicates moderate impairment, and 2 or less indicates severe functional impairment. ADL was calculated at the time of discharge in the study subjects.

Depression in the study subjects was evaluated using the Geriatric Depression Scale (GDS). The GDS is designed specifically for rating depression in the elderly and represents a reliable and valid self-rating scale for the elderly, with a sensitivity of 84% and specificity of 95% [8]. Patients scoring between 0 and 9 were labeled as having no depression, those having scores between 10 and 19 as having mild depression, and patients with scores > 20 were labeled as having severe depression. The GDS was calculated at the time of discharge in all study subjects.

Assessment of comorbid conditions was done using the Charlson Comorbidity Index (CCI). The CCI is a well-established predictor of in-hospital mortality in nonsurgical patients [9] and in those with specific diseases [10]. Each comorbidity has an associated weight (1-6) based on the adjusted risk of mortality or resource use. The sum of all the weights results in a single comorbidity score, with scores of zero depicting no comorbidity. The CCI was also calculated at the time of discharge.

Socioeconomic status was calculated using the Kuppuswamy Scale. Height and weight were measured by a stadiometer and standardized weighing machine. Height was measured to the nearest 0.1 cm and weight to the nearest 0.1 kg.

The present study was an observational, cross-sectional study conducted in the inpatient department of Internal Medicine in a tertiary care hospital.

Statistical analyses were performed using the Statistical Package for Social Sciences (SPSS) version 23 (IBM Corporation, Armonk, NY, USA). Data were described as frequencies and percentages, as well as means and standard deviations (SD). Associations between mortality in the elderly after discharge from the hospital were evaluated using the Chi-square test for binomial and Mann-Whitney U test in ordinal variables. Factors that were found to be associated with three-month mortality (<0.05) were included in the multivariate regression model to identify their independent effect. Statistical significance was set at p<0.05 in the final analysis.

## Results

Among the 177 total patients enrolled, 54.2% (96) were males and 45.8% (81) were females; 107 (60.45%) patients were between 60 and 69 years, and 70 patients (39.55%) were more than 69 years. The mean age of the study population was 68.64±7.73 years. A total of 137 (77.4%) patients belonged to the upper lower class and 40 (22.7%) patients belonged to the lower middle socioeconomic class by Kuppuswamy sociodemographic scale. Furthermore, 72 (40.68%) patients lived with their spouses, and 105 (59.32%) patients lived in joint families. Of the patients, 22.6% were malnourished and 49.7% were at risk of malnutrition. A total of 87 (49.1%) participants were addicted to either smoking or alcohol. The risk of malnutrition was seen in those elderly who were dependent on their children (p=0.005 ) and who had CCI > 3 (p<0.001 ). This shows that it is pertinent to focus on contributory factors such as demographic profiles and comorbidities of the patients while assessing the nutritional status for better and focused interventional approaches. Of the study participants, 24.29% died during the three-month follow-up. Table 1 shows the clinical characteristics of the study participants.

**Table 1:** Clinical characteristics of the study participants (N=177) *Activities of daily living (ADL) of 134 study participants at the end of three months were recorded as 43 had expired. BMI: body mass index; ADL: activities of daily living; MNA: Mini Nutritional Assessment; CCI: Charlson Comorbidity Index; GDS: Geriatric Depression Scale

Variables	Categories	Frequencies (n)	Percentages (%)
BMI (kg/m^2^)	<18.5	9	5.08
	18.5–24.9	148	83.62
	>24.9	20	11.3
Comorbidity	Diabetes	56	31.64
	HTN	64	36.16
	No comorbidity	57	32.2
Mobility status	No aid used	140	79.09
	With aid	37	20.91
ADL (at discharge)	≤4	107	60.45
	>4	70	39.55
ADL (at the end of three months)*	≤4	38	28.36
	>4	96	71.64
MNA	Well-nourished	49	27.68
	At-risk	88	49.72
	Malnourished	40	22.6
CCI	≤2	27	15.25
	3–5	83	46.90
	>5	67	37.85
GDS	Depression	49	27.68
	No depression	128	72.32

Table 2 and Table 3 show the association of various demographic and clinical parameters with mortality. Malnutrition, ADL, and Charlson Comorbidity Index were found to be associated with three-month mortality, with p values of 0.005, 0.021, and 0.017, respectively.

**Table 2:** Association of sociodemographic and parameters with three-month mortality among the study participants (N=177)

Variables	Three-month mortality			Chi-square test values	
	Dead (n (%))	Alive (n (%))	Total (n (%))	𝝌2	p value
Age					
60–69	25 (23.58%)	81 (76.42%)	106 (100%)	0.072	0.788
>69	18 (25.35%)	53 (74.65%)	71 (100%)		
Gender					
Male	27 (28.13%)	69 (71.87%)	96 (100%)	1.674	0.196
Female	16 (19.75%)	65 (80.25%)	81 (100%)		
Socioeconomic status					
Upper lower	34 (24.82%)	103 (75.18%)	137 (100%)	0.090	0.764
Lower middle	9 (22.5%)	31 (77.5%)	40 (100%)		
Caretaker					
Spouse	12 (16.67%)	60 (83.33%)	72 (100%)	3.839	0.050
Children	31 (29.52%)	74 (70.48%)	105 (100%)		
Smoking					
Present	21 (28%)	54 (72%)	75 (100%)	0.972	0.324
Absent	22 (21.57%)	80 (78.43%)	102 (100%)		
Alcohol					
Present	3 (25%)	9 (75%)	12 (100%)	-	1.000
Absent	40 (24.24%)	125 (75.76%)	165 (100%)		

**Table 3:** Association of clinical parameters with three-month mortality among the study participants (N=177) *Mann–Whitney U test was used to compare Charlson Comorbidity Index (CCI), body mass index (BMI), and Mini Nutritional Assessment (MNA) scores. BMI: body mass index; CCI: Charlson Comorbidity Index; GDS: Geriatric Depression Scale; MNA: Mini Nutritional Assessment; ADL: activities of daily living

Variables	Three-month mortality			Chi-square test value	
	Dead (n (%))	Alive (n (%))	Total (n (%))	𝝌2	p value
Diabetes					
Present	13 (23.21%)	43 (76.79%)	56 (100%)	0.052	0.820
Absent	30 (24.79%)	91 (75.21%)	121 (100%)		
Hypertension					
Present	15 (30.61%)	34 (69.39%)	49 (100%)	1.471	0.225
Absent	28 (21.88%)	100 (78.12%)	128 (100%)		
BMI^*^					
<18.5	2 (22.22%)	7 (77.78%)	9 (100%)	-	0.48
18.5­–24.9	38 (25.68%)	110 (74.32%)	148 (100%)		
>24.9	3 (15%)	17 (85%)	20 (100%)		
CCI^*^					
≤2	2 (7.41%)	25 (92.59%)	27 (100%)	2126	0.005
3–5	18 (21.69%)	65 (78.31%)	83 (100%)		
>5	23 (34.33%)	44 (65.67%)	67 (100%)		
GDS					
Depression	15 (30.61%)	34 (69.39%)	49 (100%)	1.471	0.225
No depression	28 (21.88%)	100 (78.12%)	128 (100%)		
MNA score^*^					
Well-nourished	5 (10.2%)	44 (89.8%)	49 (100%)	2138	0.006
At-risk	22 (25%)	66 (75%)	88 (100%)		
Malnourished	16 (40%)	24 (60%)	40 (100%)		
ADL (at discharge)					
≤4	36 (33.6%)	71 (66.3%)	107 (100%)	6.665	0.021
>4	7 (10%)	63 (90%)	70 (100%)		

On applying logistic regression, the malnourished group (odds ratio (OR): 3.796; p=0.025) and the at-risk group (OR: 2.315; p=0.129) were found to have higher odds for mortality. However, the CCI was not found to be a significant predictor of three-month mortality. ADL at discharge was found to be a strong predictor of three-month mortality in hospitalized elderly subjects (OR: 3.160; p=0.015). Table 4 shows the regression analysis of the various significant variables across alive and dead patients.

**Table 4:** Regression table of various significant variables across expired and alive patients CCI: Charlson Comorbidity Index; ADL: activities of daily living

Factors	Unadjusted OR	95% CI	p value	Adjusted OR	95% CI	p value
Nutritional status						
Well-nourished	-	-	-	-	-	-
At-risk	2.933	1.033–8.326	0.043	2.315	0.784–6.835	0.129
Malnourished	5.867	1.913–17.991	0.002	3.796	1.178–12.234	0.025
CCI						
≤2	-	-	-	-	-	-
3–5	3.462	0.748–16.017	0.112	1.977	0.399–9.799	0.404
>5	6.534	1.421–30.055	0.016	2.957	0.591–14.795	0.187
ADL (at discharge)						
≤4	4.563	1.897–10.978	0.001	3.160	1.256–7.952	0.015
>4	-	-	-	-	-	-
Factors	Unadjusted OR	95% CI	p value	Adjusted OR	95% CI	p value
Nutritional status						
Well-nourished	-	-	-	-	-	-
At-risk	2.933	1.033–8.326	0.043	2.315	0.784–6.835	0.129
Malnourished	5.867	1.913–17.991	0.002	3.796	1.178–12.234	0.025
CCI						
≤2	-	-	-	-	-	-
3–5	3.462	0.748–16.017	0.112	1.977	0.399–9.799	0.404
>5	6.534	1.421–30.055	0.016	2.957	0.591–14.795	0.187
ADL (at discharge)						
≤4	4.563	1.897–10.978	0.001	3.160	1.256–7.952	0.015
>4	-	-	-	-	-	-
Nutritional status						
Well-nourished	-	-	-	-	-	-
At-risk	2.933	1.033–8.326	0.043	2.315	0.784–6.835	0.129
Malnourished	5.867	1.913–17.991	0.002	3.796	1.178–12.234	0.025
CCI						
≤2	-	-	-	-	-	-
3–5	3.462	0.748–16.017	0.112	1.977	0.399–9.799	0.404
>5	6.534	1.421–30.055	0.016	2.957	0.591–14.795	0.187
ADL (at discharge)						
≤4	4.563	1.897–10.978	0.001	3.160	1.256–7.952	0.015
>4	-	-	-	-	-	-

## Discussion

This study was aimed to find whether nutritional status at admission measured using Mini Nutritional Assessment and activity of daily living (ADL) evaluated using Katz Index of Independence in Activities of Daily Living assessed at baseline and at discharge were predictors of three-month mortality in elderly hospitalized subjects. The study revealed that a definitive association does exist with both parameters. The study also showed that about half of these elderly subjects are at risk of malnutrition and one-fourth are severely malnourished. Those patients who were at risk of malnutrition did not show increased mortality when compared to well-nourished patients. Among the subjects who had ADL ≤ 4 at discharge, the risk of three-month mortality was significantly higher (p=0.021).

The prevalence of malnutrition in our study was comparable to the other Indian studies such as the one conducted by Lahiri et al. (community-based study), wherein he found that 29% of the geriatric population were malnourished, 60.4% were at-risk, and 10.2% were well-nourished [11]. This was in contrast to studies from the West, which showed that the Western population had far better nutritional status as compared to our population. The study conducted by Formiga et al. in Spain had 3.7% subjects as malnourished, 30.4% as at-risk, and 65.8% as well-nourished [12].

Tsai et al. in Taiwan conducted a large population-based longitudinal study that helped in deriving the relationship of malnutrition in predicting mortality [13]. The study enrolled 2,872 subjects and used Cox regression analysis and the net reclassification improvement (NRI) to quantify the indicators and the MNA and ADL to predict four-year mortality. Both MNA and ADL presented good independent predictive abilities in four-year mortality (p<0.001 for both). Sharma et al. conducted a large prospective observational study evaluating the association between nutritional status and readmission or death in medical inpatients aged 65 and above using the Patient-Generated Subjective Global Assessment (PG-SGA) to assess nutrition. Malnutrition was present, with a significantly higher risk of readmissions and death both within seven days (p≤0.001) and 180 days (p=0.007) [14]. All these abovementioned studies have reestablished the same fact that nutrition does serve as an important predictor of mortality in the elderly population. Through the present study, we identify the third criteria of patients who are “at risk of malnutrition.” It is crucial that physicians (geriatricians and non-geriatricians) must target this group while assessing and treating them so as to prevent the further risk of malnutrition and avert possible consequences. For these reasons, nutritional assessment is a vital part of the comprehensive assessment of geriatric patients. Furthermore, malnutrition, being a modifiable risk factor, can be easily targeted by governments and health professionals to dramatically reduce the burden in the healthcare sector attributable to sick elderly, especially with the ones with a poor nutritional status.

The study conducted by Torisson et al. on the importance and added value of functional impairment to predict mortality in 2016 compared a set of clinical risk factors and found that impairment in ADL was a stronger predictor of all-cause mortality. Hence, implementing quantitative ADL measurements could enable better individual care for the elderly in terms of outcome and mortality [15]. In another study done by Pietiläinen et al. on premorbid functional status as a predictor of one-year mortality and functional status in intensive care unit (ICU) patients aged 80 years or older, they found that a poor pre-functional status predicted an increased risk of in-hospital death (adjusted OR: 1.50; 95% CI: 1.07-2.10) and one-year mortality (OR: 2.18; 95% CI: 1.67-2.85). Among elderly ICU patients who survived after one year, they had a functional status comparable to the premorbid situation. The study concluded that a poor pre-functional status doubled the odds of death within a year [16]. These results indicate that functional status in an elderly patient coming to a hospital serves a vital role in predicting their short-term mortality during and after their hospital stay, which is why it is of paramount importance to focus on the assessment and interventional approaches to improve the functional status in these categories of patients.

Limitations

This is an observational study conducted on hospitalized elderly, which might not be a proper representation of the overall geriatric population. Hence, to project this data to the general population, we will need to conduct further studies with larger samples in the non-hospitalized elderly population. Few of the study participants did not complete the study due to a lengthy questionnaire; hence, it is possible that eligible participants were missed. Therefore, a more comprehensive and compact questionnaire could be used in future studies to avoid this problem. Also, patients who were medically unstable or incomprehensible were excluded from the study. These patients might represent those elderly that are likely to be at risk of malnutrition as a consequence of reduced intake due to their ongoing ailments. The prevalence of malnutrition is more likely to increase with age and declining functional ability. We are not sure if the observed prevalence of malnutrition from the study was a cause or a possible consequence of the observed decline in functional status and the three-month all-cause mortality outcomes at follow-up [17]. This study has not used other indexes that have been used previously in the elderly, such as the Modified Frailty Index [18]. However, taking into view the results of the current study, stating that both nutritional and functional status are clinically significant parameters to predict mortality outcomes in the elderly age group, there is a need of further exploration in future studies, especially while formulating for some interventional measures.

## Conclusions

To summarize, as the elderly population is often easily predisposed to numerous comorbidities and ailments, it is very important to focus on their nutritional and functional abilities since there is a decline in these aspects, which affects their mortality outcomes. This study provides data that improving patient nutrition and ADL at discharge will help in better outcomes of patient care in terms of mortality benefits. These findings reinforce that when discharging elderly patients from the hospital, normalization of biochemical parameters is not only sufficient; optimization of nutritional status and ADL is also required. Further research is, however, recommended to project these findings to a larger population and also to formulate interventional strategies and assess their impact on the outcomes among this group of population.

## References

[1] Drewnowski A, Evans WJ (2001). Nutrition, physical activity, and quality of life in older adults: summary. J Gerontol A Biol Sci Med Sci.

[2] Skipper A (2012). Agreement on defining malnutrition. JPEN J Parenter Enteral Nutr.

[3] Prell T, Perner C (2018). Disease specific aspects of malnutrition in neurogeriatric patients. Front Aging Neurosci.

[4] Reuben DB, Rubenstein LV, Hirsch SH, Hays RD (1992). Value of functional status as a predictor of mortality: results of a prospective study. Am J Med.

[5] Middleton MH, Nazarenko G, Nivison-Smith I, Smerdely P (2001). Prevalence of malnutrition and 12-month incidence of mortality in two Sydney teaching hospitals. Intern Med J.

[6] Vellas B, Guigoz Y, Garry PJ, Nourhashemi F, Bennahum D, Lauque S, Albarede JL (1999). The Mini Nutritional Assessment (MNA) and its use in grading the nutritional state of elderly patients. Nutrition.

[7] Patricia P (2003). Katz for the Association of Rheumatology Health Professionals Outcomes Measures Task Force: measures of adult general functional status: the Barthel Index, Katz Index of Activities of Daily Living, Health Assessment Questionnaire (HAQ), MACTAR Patient Preference Disability Questionnaire, and Modified Health Assessment Questionnaire (MHAQ). Arthritis Care Res.

[8] JA Yesavage (19862137). The use of self-rating depression scales in the elderly. Handbook for clinical memory assessment of older adults. Poon LW, Crook T, Davis KL, Eisdorfer C, Gurland BJ, Kaszniak AW, Thompson LW (ed.

[9] Sundararajan V, Henderson T, Perry C, Muggivan A (2004). Quan H, Ghali WA: New ICD-10 version of the Charlson comorbidity index predicted in-hospital mortality. J Clin Epidemiol.

[10] Zavascki AP, Fuchs SC (2007). The need for reappraisal of AIDS score weight of Charlson comorbidity index. J Clin Epidemiol.

[11] Lahiri S, Biswas A, Santra S (2015). Assessment of nutritional status among elderly population in a rural area of West Bengal, India. Int J Med Sci Public Health.

[12] Formiga F, Ferrer A, de Ulíbarri Pérez JI Detecting malnutrition and predicting mortality in the Spanish oldest old: Utility of the Controlling Nutritional Status (CONUT) score compared with the Mini Nutritional Assessment (MNA) score. Eur Geriatr.

[13] Tsai AC, Lee LC, Wang JY (2013). Complementarity of the Mini-Nutritional Assessment and Activities of Daily Living for predicting follow-up mortality risk in elderly Taiwanese. Br J Nutr.

[14] Sharma Y, Miller M, Kaambwa B, Shahi R, Hakendorf P, Horwood C, Thompson C (2017). Malnutrition and its association with readmission and death within 7 days and 8-180 days postdischarge in older patients: a prospective observational study. BMJ Open.

[15] Torisson G, Stavenow L, Minthon L, Londos E (2017). Importance and added value of functional impairment to predict mortality: a cohort study in Swedish medical inpatients. BMJ Open.

[16] Pietiläinen L, Hästbacka J, Bäcklund M, Parviainen I, Pettilä V, Reinikainen M (2018). Premorbid functional status as a predictor of 1-year mortality and functional status in intensive care patients aged 80 years or older. Intensive Care Med.

[17] Griffin A, O'Neill A, O'Connor M, Ryan D, Tierney A, Galvin R (2020). The prevalence of malnutrition and impact on patient outcomes among older adults presenting at an Irish emergency department: a secondary analysis of the OPTI-MEND trial. BMC Geriatr.

[18] Bhurchandi S, Kumar S, Agrawal S, Acharya S, Jain S, Talwar D, Lomte S (2021). Correlation of sarcopenia with modified frailty index as a predictor of outcome in critically ill elderly patients: a cross-sectional study. Cureus.

